# The Local Application of the Subuitrate of Bismuth to Prevent Pitting in Variola

**Published:** 1866-03

**Authors:** 


*The local application of the Subuitrate of Bismuth to prevent pitting in Variola.*—In the American Journal of the Medical Sciences Dr. Wm. R. Hamilton, of St. Augustine, Ill., reports two cases of Variola in which he prevented pitting by the daily application of the Subuitrate of Bismuth and a black mask. In the first case he applied the Bismuth and Creta Preparata in equal parts, first lubricating the face with sweet oil; the application was made twice a day, the face being covered with a black mask. The eruption passed through its regular course, leaving but few scars perceptible on the face, the only part to which the application was made.

In the second case the eruption was confluent upon the hands and face. Dr. H. says: "There appeared to be but one pock from his eyes to his chin, as well as on the dorsal aspect of his hands." The Subuitrate of Bismuth, pure, was applied to the face and hands twice a day, after lubricating the parts with sweet oil, as in the other case. The face was covered with a black mask; his hands were left uncovered. There is scarcely a pit to be found on the face; not one is perceptible on the hands.

